# A Novel Clinical Score Integrating Low‐Voltage Zones and Biomarkers Predicts Atrial Fibrillation Recurrence Post‐Ablation

**DOI:** 10.1002/clc.70218

**Published:** 2025-11-12

**Authors:** Ying Han, Jingzhe Liu, Xiaobo Liu, Hanyue Zheng, Juan Wang, Juan Zhang

**Affiliations:** ^1^ Department of Cardiology The Second Qilu Hospital of Shandong University Jinan Shandong China; ^2^ Department of Critical Care Medcine Shandong Public Health Clinical Center Jinan Shandong China; ^3^ Shandong Blood Center Jinan Shandong China

**Keywords:** atrial fibrillation recurrence, catheter ablation, low‐voltage zone, red cell distribution width, risk stratification

## Abstract

**Purpose:**

Despite technological advances, predicting atrial fibrillation (AF) recurrence after catheter ablation remains a clinical challenge. We developed a novel multi‐parametric model integrating electrophysiological substrate characteristics, structural remodeling, and inflammatory/metabolic biomarkers to improve risk stratification.

**Methods:**

This retrospective study analyzed 279 consecutive patients undergoing first‐time AF ablation (June 2022 to January 2024) with 12‐month follow‐up. Using a 7:3 training‐validation split, we identified independent predictors through multivariate logistic regression.

**Results:**

Four key parameters emerged as powerful predictors: low‐voltage zone extent (LVZ), high‐sensitivity C‐reactive protein (hs‐CRP), red cell distribution width (RDW), and left atrial diameter (LAD). The composite model showed exceptional discrimination (AUC, in the training set and 0.84 in the validation set), significantly outperforming both individual parameters (LAD AUC 0.77, LVZ 0.75) and the APPLE score (AUC: 0.73, *p* < 0.001). The model stratified patients into five distinct risk categories (recurrence risk < 5% to > 70%) with strong clinical utility.

**Conclusion:**

This is the first East Asian study to integrate voltage mapping with hematological‐inflammatory biomarkers, providing a cost‐effective and precise tool for post‐ablation management. The model's performance and generalizability support its adoption in precision medicine pathways, particularly for guiding substrate modification in high‐risk patients.

## Graphical abstract

A novel clinical score integrating low‐voltage zone extent (LVZ), high‐sensitivity C‐reactive protein (hs‐CRP), red cell distribution width (RDW), and left atrial diameter (LAD) predicts atrial fibrillation recurrence post‐ablation. It enables personalized postprocedural management by stratifying patients into five distinct risk categories.

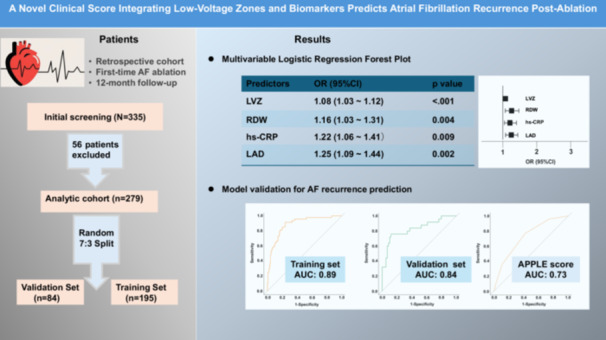

**Abbreviations**
AF: atrial fibrillation
CABG: coronary artery bypass grafting
ECG: electrocardiogram
eGFR: estimated glomerular filtration rate
HDL‐C: high‐density lipoprotein cholesterol
hs‐CRP: high‐sensitivity C‐reactive protein
ICE: intracardiac echocardiography
LAD: left atrial diameter
LDL‐C: low‐density lipoprotein cholesterol
LVEDD: left ventricular end‐diastolic diameter
LVEF: left ventricular ejection fraction
LVESD: left ventricular end‐systolic diameter
LVZ: low‐voltage zone
NT‐proBNP: N‐terminal pro‐B‐type natriuretic peptide
RDW: red cell distribution width

## 1 Introduction

Atrial fibrillation (AF) has become a global epidemic, currently affecting 1%−2% of the population worldwide. Projections suggest its prevalence will increase 2.5‐fold by 2050 [1, 2]. This arrhythmia carries significant clinical consequences, including a fivefold higher stroke risk and threefold increased cardiovascular mortality. Although catheter ablation achieves acute success rates of 70%−80% as first‐line therapy, long‐term outcomes remain suboptimal with 30%−50% recurrence rates [3, 4]. Post‐ablation AF recurrence correlates with poor clinical outcomes and represents a major challenge in electrophysiology practice [5].

Multiple patient factors influence recurrence risk, including female sex, advanced age, cardiovascular risk factors, left ventricular dysfunction, epicardial adipose tissue burden, myocardial fibrosis, and atrial dilatation [6, 7, 8]. Existing research often examines isolated mechanisms like metabolic markers or structural remodeling, overlooking their complex interactions [9, 10]. Current scoring systems (APPLE, MB‐LATER) show limited predictive value [11, 12, 13].

This study advances beyond conventional approaches by integrating three key domains: electrophysiological substrates (low‐voltage zones, LVZs), structural remodeling (LAD), and inflammatory/metabolic markers (high‐sensitivity C‐reactive protein [hs‐CRP] and red cell distribution width [RDW]). We present a novel multidimensional framework for risk stratification and clinical decision‐making. Our findings address critical knowledge gaps and offer a generalizable model for precision medicine in AF management, with significant potential to improve patient outcomes.

## 2 Materials and Methods

### 2.1 Study Design and Participants

This retrospective cohort study evaluated consecutive patients undergoing first‐time catheter ablation for AF at the Second Hospital of Shandong University between June 2022 and January 2024. Following comprehensive preprocedural counseling that detailed the intervention's objectives, potential risks, and expected outcomes, all participants provided written informed consent. From the 335 initially screened patients, we excluded 56 individuals (16.7%) meeting predefined exclusion criteria. The remaining 279 patients comprised our final study cohort. These participants were subsequently stratified by documented AF late recurrence status (defined as any ECG‐confirmed episode lasting ≥ 30 s occurring between 3 and 12 months post‐ablation). Inclusion criteria: (1) AF confirmed by 12‐lead ECG, (2) age 18‐80 years, and (3) ablation using 3D electroanatomic mapping. Exclusion criteria included: incomplete data, prior ablation, severe comorbidities (eGFR< 30 mL/min/1.73 m², active infections, advanced peripheral artery disease), uncontrolled thyroid dysfunction, significant structural heart disease (severe valvulopathy, valve replacement, CABG), or pregnancy. The study protocol was approved by the Institutional Review Board of the Second Hospital of Shandong University (KYLL‐2021[KJ]P‐0349) and prospectively registered with the Chinese Clinical Trial Registry (ChiCTR2400094772).

### 2.2 Diagnostic Criteria and Outcome Definitions

This study adopted the 2023 ACC/AHA/ESC guideline definitions for AF classification and recurrence [14]. Paroxysmal AF was defined as recurrent, self‐terminating episodes (typically lasting < 7 days) or those requiring intervention for termination, while persistent AF referred to sustained episodes (> 7 days) requiring pharmacologic or electrical cardioversion to restore sinus rhythm, including those terminated by intervention after ≥ 7 days of continuous arrhythmia. For outcome assessment, late recurrence was defined as any documented rapid atrial arrhythmia (AF, flutter, or tachycardia) lasting ≥ 30 s on 12‐lead ECG or Holter monitoring occurring beyond the 3‐month blanking period. Early recurrences (≤ 3 months post‐ablation) were excluded as potential transient phenomena.

### 2.3 Clinical Data Collection

Trained personnel collected baseline clinical and echocardiographic data through standardized medical record review. Cardiac structural parameters included left atrial diameter (LAD), left ventricular ejection fraction (LVEF), left ventricular end‐diastolic diameter (LVEDD), and left ventricular end‐systolic diameter (LVESD), all measured according to current echocardiography guidelines. Preoperative laboratory evaluation encompassed: (1) hematologic profiles (red blood cell count, RDW, lymphocyte count, etc); (2) coagulation profile (d‐dimer, fibrinogen); (3) metabolic biomarkers (liver function, renal function, and lipid panel); and (4) inflammatory markers (hs‐CRP). All blood samples were collected after standardized fasting and analyzed using validated methods.

### 2.4 Catheter Ablation Protocol

All patients underwent preprocedural transesophageal echocardiography to rule out intracardiac thrombi. Under local anesthesia (1% lidocaine), bilateral femoral venous access was obtained. Systemic heparinization maintained ACT 300−350 s. Using intracardiac echocardiography (ICE) guidance, we performed transseptal puncture and created 3D electroanatomic maps (CARTO 3 system) of the left atrium and pulmonary veins. High‐density voltage mapping (Pentaray NAV catheter) characterized the atrial substrate. Point‐by‐point radiofrequency ablation (ThermoCool SmartFlex catheter) targeted ablation index 400−550. Following pulmonary vein isolation, additional linear lesions (roof/mitral isthmus) were created for persistent AF or induced tachycardia, with bidirectional block confirmed by pacing. For patients who failed to achieve sinus rhythm following ablation, synchronized electrical cardioversion (100−200 J, biphasic shock) was administered.

### 2.5 Electroanatomic Voltage Mapping Protocol

Post‐ablation high‐density voltage mapping was conducted using a Pentaray catheter during sinus rhythm, with catheter‐tissue contact verified by impedance monitoring (CARTO 3 TPI system). We applied standardized voltage thresholds: dense scar (< 0.1 mV, red), LVZs (0.1−0.5 mV, orange‐blue gradient), and normal myocardium (> 0.5 mV, purple). Electroanatomic abnormalities were defined as areas < 0.5 mV. Fibrotic burden was quantified as LVZ to total atrial surface area ratio using CARTO 3's Area Measurement tool. Two blinded operators independently performed measurements, demonstrating excellent interobserver agreement.

### 2.6 Follow‐Up

Patients underwent scheduled evaluations at 1, 3, 6, and 12 months post‐ablation, combining clinic visits, telephone interviews, and hospital records. Each assessment included 24‐hour Holter monitoring. We instructed patients to promptly report arrhythmia symptoms. The primary endpoint was ECG‐documented atrial tachyarrhythmia (AF, flutter, or tachycardia) lasting ≥ 30 s after the 3‐month blanking period, verified by 24‐hour ambulatory Holter monitoring or 12‐lead ECG.

### 2.7 Model Development and Validation Approach

We employed computer‐generated block randomization (seed: 1982; 7:3 ratio) to create balanced training (*n* = 195) and internal validation (*n* = 84) cohorts. This split was designed to prioritize statistical power for developing a stable multivariable model in the derivation phase, while retaining a sufficiently sized cohort for initial internal validation. All model development—including feature selection, parameter tuning, and algorithm optimization—was exclusively performed on the training set. The validation set remained strictly isolated until final testing to ensure unbiased performance assessment (Figure S1). This approach aligns with established methodological recommendations for internal validation of clinical prediction models of a similar scale [15].

### 2.8 Statistical Analysis

Analyses were conducted using SPSS 25.0. Continuous variables were tested for normality (Shapiro−Wilk test) and presented as mean ± SD (*t*‐test) or median (interquartile range [IQR]) (Mann−Whitney *U* test). Categorical variables were compared using *χ*² or Fisher's exact tests. Univariate logistic regression identified potential predictors, with significant variables (*p* < 0.05) entering multivariate analysis. The final model incorporated LVZ%, hs‐CRP, RDW, and LAD. Model performance was evaluated through receiver operating characteristic (ROC) curve analysis, with area under the curve (AUC) calculations for both individual parameters and the composite model. Model calibration was assessed using the Hosmer−Lemeshow goodness‐of‐fit test, while clinical utility was evaluated through decision curve analysis (DCA). DeLong's test was applied to evaluate whether the difference in AUCs between the two models was statistically significant. Continuous predictors were dichotomized using ROC‐derived cutoffs. Regression coefficients were converted to integer scores, creating a 5‐tier risk stratification system (< 5% to > 70% recurrence probability) aligned with clinical decision points.

## 3 Results

### 3.1 Baseline Demographic and Clinical Characteristics

Of the 335 initially screened patients, 56 were excluded (prior ablation, *n* = 4; valvular AF, *n* = 5; missing voltage data, *n* = 2; incomplete records, *n* = 10; uncontrolled thyroid dysfunction, *n* = 1; lost to follow‐up, *n* = 24), yielding 279 patients for analysis (Figure S3). The cohort had a median age of 66 years (IQR 59−72), with 43.4% females. AF subtypes included paroxysmal (62.0%) and non‐paroxysmal (38.0%). Common comorbidities were hypertension (54.8%), diabetes (19.5%), dyslipidemia (15.8%), coronary artery disease (59.5%), and prior cerebrovascular events (15.1%). Further details are presented in Table 1.

**Table 1:** Baseline characteristics of 279 AF patients.

**Characteristics**	**Total (*n*** = **279)**	**Non‐recurrence group (*n*** = **206)**	**Recurrence group (*n*** = **73)**	***p* value**
Age (years)	66.00 (59.00, 72.00)	65.00 (58.00, 71.00)	69.00 (63.00, 72.00)	**0.022**
Female, *n* (%)	121 (43.37)	87 (42.23)	34 (46.58)	0.520
Non‐paroxysmal AF, *n* (%)	106 (37.99)	64 (31.07)	42 (57.53)	**< 0.001**
AF duration, months	15.00 (9.00, 30.00)	12.00 (7.05, 30.00)	24.00 (12.00, 36.00)	**0.012**
Smoking, *n* (%)	96 (34.41)	75 (36.41)	21 (28.77)	0.238
Drinking, *n* (%)	88 (31.54)	60 (29.13)	28 (38.36)	0.145
Hypertension, *n* (%)	153 (54.84)	115 (55.83)	38 (52.05)	0.578
Diabetes mellitus, *n* (%)	54 (19.35)	33 (16.02)	21 (28.77)	**0.018**
Hyperlipidemia, *n* (%)	44 (15.77)	29 (14.08)	15 (20.55)	0.192
Stroke, *n* (%)	42 (15.05)	26 (12.62)	16 (21.92)	0.056
Coronary heart disease, *n* (%)	166 (59.50)	116 (56.31)	50 (68.49)	0.068
LAD (mm)	42.00 (39.00, 45.00)	41.00 (39.00, 43.00)	45.00 (42.00, 48.00)	**< 0.001**
LVEDD (mm)	47.00 (45.00, 50.00)	47.00 (45.00, 50.00)	47.00 (44.00, 50.00)	0.744
LVESD (mm)	31.00 (29.00, 34.00)	31.00 (30.00, 34.00)	31.00 (28.00, 34.00)	0.467
LVEF (%)	0.60 (0.57, 0.64)	0.60 (0.57, 0.65)	0.60 (0.57, 0.63)	0.335
Erythrocytes (10^9^/L)	4.61 ± 0.58	4.59 ± 0.57	4.69 ± 0.60	0.211
Hemoglobin (g/L)	141.01 ± 17.11	140.57 ± 16.99	142.25 ± 17.50	0.472
RDW (fL)	43.20 (40.85, 45.10)	42.70 (40.60, 44.60)	44.70 (42.80, 46.60)	**< 0.001**
Leukocytes (10^9^/L)	6.05 (4.98, 7.09)	6.08 (5.04, 7.17)	6.05 (4.96, 6.90)	0.613
Neutrophils (10^9^/L)	3.58 (2.91, 4.51)	3.55 (2.92, 4.51)	3.64 (2.66, 4.47)	0.806
Lymphocytes (10^9^/L)	1.71 (1.34, 2.04)	1.73 (1.37, 2.14)	1.66 (1.34, 1.86)	0.194
Monocytes (10^9^/L)	0.45 (0.35, 0.55)	0.45 (0.35, 0.54)	0.47 (0.39, 0.58)	0.117
Platelets (10^9^/L)	212.05 ± 47.91	209.41 ± 49.66	219.49 ± 42.00	0.095
TG (mmol/L)	1.19 (0.90, 1.60)	1.17 (0.90, 1.58)	1.22 (0.91, 1.68)	0.788
TC (mmol/L)	3.98 (3.45, 4.70)	4.05 (3.52, 4.65)	3.85 (3.20, 4.81)	0.179
LDL‐C (mmol/L)	2.26 (1.71, 2.79)	2.30 (1.77, 2.78)	2.16 (1.63, 2.90)	0.400
HDL‐C (mmol/L)	1.14 (0.96, 1.37)	1.15 (0.96, 1.42)	1.11 (0.96, 1.27)	0.176
Albumin (g/L)	42.55 ± 3.47	42.58 ± 3.63	42.47 ± 3.01	0.784
Hcy (μmol/L)	15.80 (12.10, 17.95)	15.85 (12.40, 18.15)	14.20 (11.80, 17.80)	0.093
Cys‐C (mg/L)	1.16 (1.03, 1.31)	1.15 (1.03, 1.29)	1.18 (1.03, 1.36)	0.556
hs‐CRP (mg/L)	2.60 (1.20, 5.15)	2.27 (1.10, 4.10)	5.20 (1.80, 7.10)	**< 0.001**
Uric acid (μmol/L)	338.11 ± 97.32	340.19 ± 99.97	332.23 ± 89.83	0.549
Creatinine (μmol/L)	70.00 (62.00, 79.00)	71.00 (62.00, 79.00)	70.00 (64.00, 76.00)	0.830
eGFR (mL/min)	87.27 ± 22.11	87.49 ± 22.33	86.64 ± 21.61	0.776
NT‐proBNP (pg/mL)	509.00 (302.50, 872.10)	476.35 (261.12, 791.45)	613.80 (351.90, 1175.00)	**0.018**
d‐dimer (ug/mL)	0.27 (0.17, 0.43)	0.28 (0.17, 0.41)	0.25 (0.18, 0.52)	0.614
Fibrinogen (g/L)	2.93 (2.61, 3.29)	2.94 (2.61, 3.29)	2.88 (2.57, 3.28)	0.607
Total left atrial area (cm²)	193.80 (167.80, 224.90)	183.80 (165.30, 221.52)	213.80 (187.10, 231.20)	**< 0.001**
LVZ (%)	19.09 (9.68, 29.78)	15.27 (8.32, 24.31)	28.31 (20.48, 34.17)	**< 0.001**
LVZ				**< 0.001**
< 10%, *n* (%)	75 (26.88)	72 (34.95)	3 (4.11)	
10%−20%, *n* (%)	71 (25.45)	60 (29.13)	11 (15.07)	
20%−30%, *n* (%)	64 (22.94)	39 (18.93)	25 (34.25)	
> 30%, *n* (%)	69 (24.73)	35 (16.99)	34 (46.58)	

*Note:* Data are presented as *n* (%), mean ± standard deviation, or median (interquartile range), as appropriate. *p* values for the comparison between the recurrence and non‐recurrence groups were derived from the Student's *t*‐test (for normally distributed continuous variables), Mann−Whitney *U* test (for skewed continuous variables), or chi‐square test (for categorical variables), as appropriate. A two‐sided *p* < 0.05 was considered statistically significant. Bold values indicate a statistically significant difference *p* < 0.05.

Abbreviations: AF, atrial fibrillation; Cys‐C, cystatin C; eGFR, estimated glomerular filtration rate; Hcy, homocysteine; HDL‐C, high‐density lipoprotein cholesterol; hs‐CRP, high‐sensitivity C‐reactive protein; LAD, left atrial diameter; LDL‐C, low‐density lipoprotein cholesterol; LVEDD, left ventricular end‐diastolic diameter; LVEF, left ventricular ejection fraction; LVESD, left ventricular end‐systolic diameter; LVZ, low‐voltage zone; NT‐proBNP, N‐terminal pro‐B‐type natriuretic peptide; RDW, red cell distribution width; TC, total cholesterol; TG, triglyceride.

### 3.2 Cohort Balance Validation

The study population was divided into a training cohort (*n* = 195) and a validation cohort (*n* = 84), with both groups demonstrating well‐balanced baseline characteristics overall. While minor statistical differences were detected in serum creatinine (*p *= 0.019) and fibrinogen levels (*p *= 0.014), these variations were deemed clinically insignificant and unlikely to impact the analysis. Importantly, all other measured variables, including demographics, comorbidities, and AF subtypes, showed no significant differences (*p *> 0.05) between the two cohorts. These findings confirm that the training and validation groups were appropriately matched, supporting the robustness of subsequent analyses. Further details are provided in Table S4.

### 3.3 Differential Clinical Features Between Recurrence Groups

**Figure 1:**
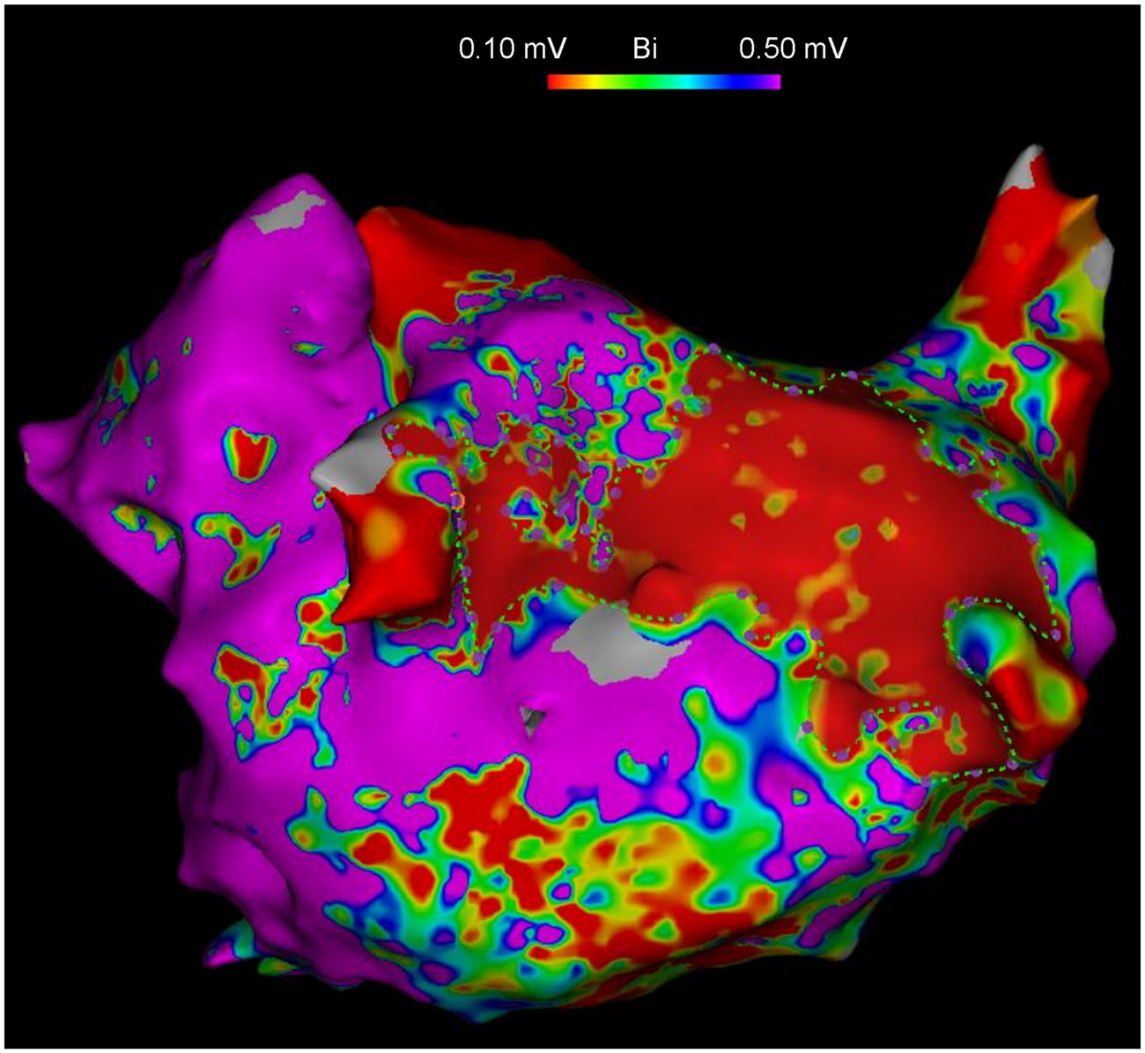
Representative high‐density voltage mapping of the left atrium (LA) demonstrating the distribution of low voltage zones (LVZs). The color‐coded voltage map delineates: dense scar tissue (< 0.1 mV, red), LVZs (0.1–0.5 mV, orange‐to‐blue gradient), and normal myocardium (> 0.5 mV, purple).

Comparative analysis between recurrence (*n* = 73) and non‐recurrence (*n* = 206) groups demonstrated comparable distributions (all *p* > 0.05) for demographic factors (sex distribution), cardiovascular risk profiles (hypertension, dyslipidemia, coronary disease, stroke history), lifestyle factors (tobacco and alcohol use), cardiac structural parameters (LVEF, LVEDD, LVESD), hematologic indices (complete blood count components), metabolic markers (lipid profiles, renal function tests), and coagulation parameters (d‐dimer, fibrinogen). However, the recurrence group was significantly older (median 69 vs. 65 years, *p* = 0.022), had longer AF duration (24 vs. 12 months, *p* = 0.012), higher prevalence of diabetes (28.8% vs. 16.0%, *p* = 0.018), and a greater proportion of non‐paroxysmal AF (57.5% vs. 31.1%, *p*< 0.001). Quantitative comparisons demonstrated that patients with recurrence exhibited larger LAD (45 vs. 41 mm, *p* < 0.001), higher RDW (44.7 vs. 42.7 fL, *p* < 0.001), elevated hs‐CRP levels (5.20 vs. 2.27 mg/L, *p* < 0.001), increased NT‐proBNP concentrations (613.8 vs. 476.4 pg/mL, *p* = 0.018), greater total left atrial area (213.8 vs. 183.8 cm², *p* < 0.001), and more extensive LVZs (28.3% vs. 15.3%, *p *< 0.001). A clear dose–response relationship emerged between LVZ extent and recurrence rates (*p *< 0.001), with the highest recurrence rate observed in patients with > 30% LVZ involvement (46.6%) compared to just 4.1% recurrence in those with < 10% involvement, highlighting the critical role of atrial substrate characteristics in predicting procedural outcomes (Figure 1).

### 3.4 Risk Factors for AF Recurrence Post‐Ablation

**Figure 2:**
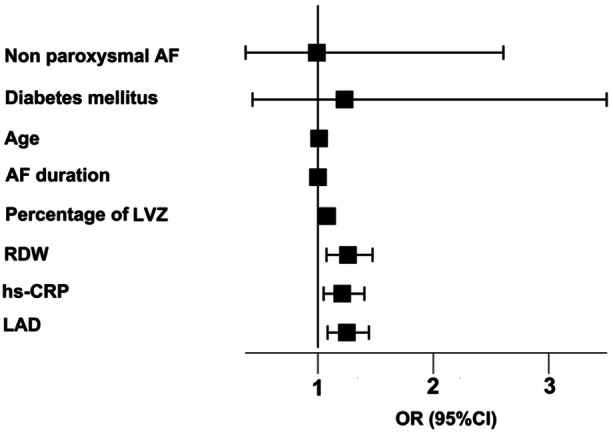
Forest plot of multivariable logistic regression analysis for predictors in the training cohort. The continuous variables (LVZ, RDW, hs‐CRP, and LAD) were modeled as linear terms. The odds ratios (OR) represent the change in risk per one‐unit increment (e.g., per 1% for LVZ, per 1 fL for RDW, per 1 mg/L for hs‐CRP, and per 1 mm for LAD).

Initial univariate analysis of the training cohort (*n* = 195) identified multiple significant predictors of AF recurrence (all *p* < 0.05), including AF type (non‐paroxysmal vs paroxysmal), diabetes mellitus, advanced age, LVZ extent, RDW, hs‐CRP, and increased LAD (Table 2). Subsequent multivariate logistic regression analysis refined these findings, revealing four robust independent predictors of AF recurrence as visually represented in the forest plot (Figure 2): LVZ extent (OR 1.08, 95% CI 1.03−1.12; *p* < 0.001), RDW (OR 1.16, 1.03−1.31; *p* = 0.004), hs‐CRP (OR 1.22, 1.06−1.41; *p* = 0.009), and LAD (OR 1.25, 1.09−1.44; *p* = 0.002). Notably, all four factors retained statistical significance in the fully adjusted model, demonstrating their independent clinical value for predicting post‐ablation AF recurrence. These findings collectively highlight the interplay between structural atrial remodeling (LVZ extent, LAD), systemic inflammation (hs‐CRP), and hematologic alterations (RDW) in determining ablation outcomes.

**Table 2:** Univariable and multivariable logistic regression analysis for AF recurrence after ablation in the training set (*n* = 195).

Variables	Univariate		multivariate	
	OR (95% CI)	*p* value	OR (95% CI)	*p* value
Non‐paroxysmal AF	4.27 (2.14−8.50)	**< 0.001**	1.03 (0.39−2.71)	0.978
Diabetes mellitus	2.22 (1.07−4.62)	**0.032**	1.45 (0.53−3.98)	0.696
Age (per 1‐year increase)	1.05 (1.01−1.10)	**0.014**	1.03 (0.97−1.09)	0.659
AF duration (per 1‐month increase)	1.01 (0.99−1.03)	0.220	1.00 (0.97−1.02)	0.794
LVZ percentage (per 1% increase)	1.09 (1.05−1.12)	**< 0.001**	1.08 (1.03−1.12)	**< 0.001**
RDW (per 1 fL increase)	1.29 (1.15−1.44)	**< 0.001**	1.16 (1.03−1.31)	**0.004**
hs‐CRP (per 1 mg/L increase)	1.34 (1.17−1.53)	**< 0.001**	1.22 (1.06−1.41)	**0.009**
LAD (per 1 mm increase)	1.32 (1.18−1.46)	**< 0.001**	1.25 (1.09−1.44)	**0.002**

*Note:* All continuous variables were analyzed as linear terms in the regression models. The OR for these variables corresponds to the change in risk associated with a one‐unit increment, as specified in the variable name. Bold values indicate a statistically significant difference *p* < 0.05.

Abbreviations: hs‐CRP, high‐sensitivity C‐reactive protein; LAD, left atrial diameter; LVZ, low‐voltage zone; RDW, red cell distribution width.

### 3.5 Predictive Performance of Recurrence Risk Factors and Model Validation

**Figure 3:**
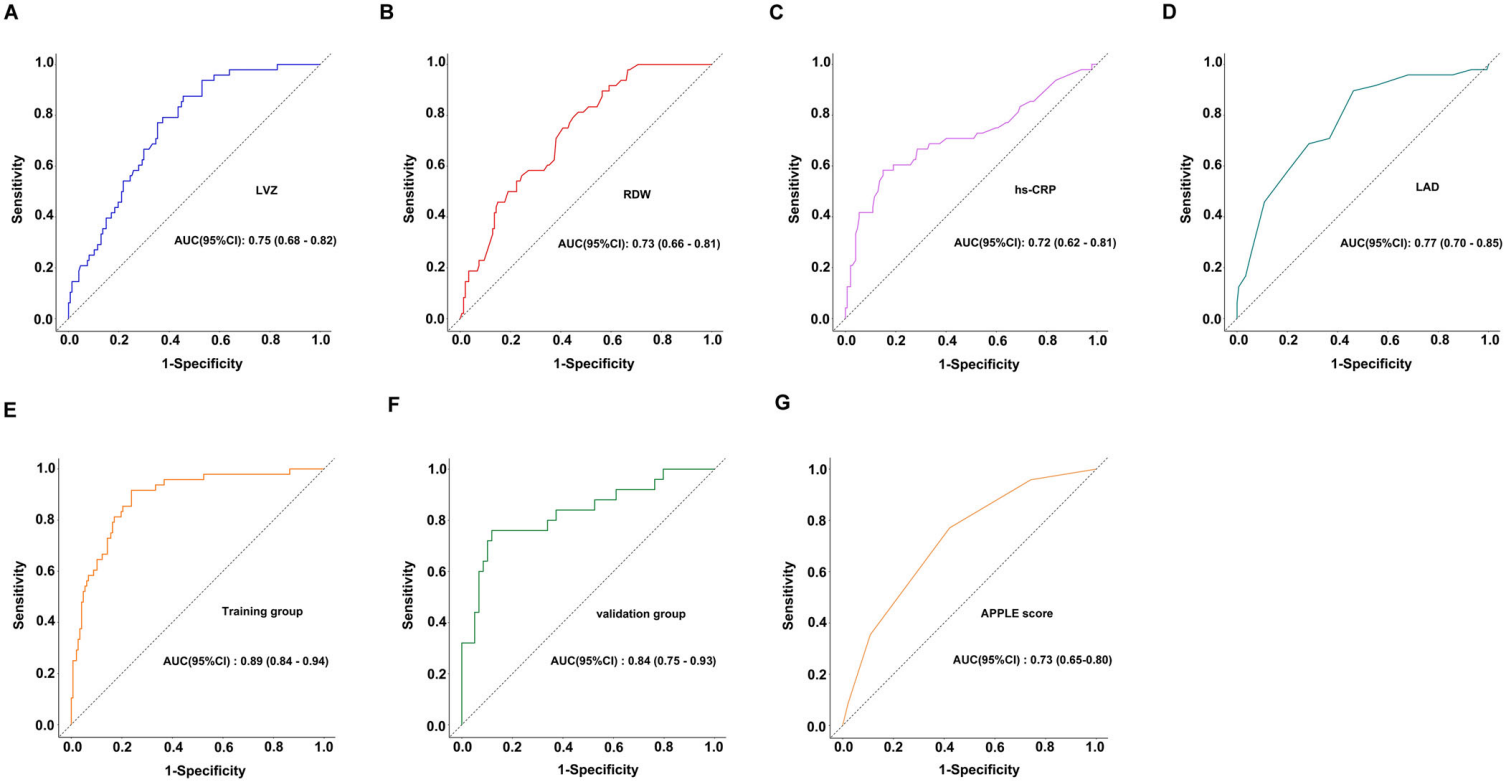
Receiver operating characteristic (ROC) curves for different predictors and models. (A) Low‐voltage zone (LVZ). (B) Red cell distribution width (RDW). (C) High‐sensitivity C‐reactive protein (hs‐CRP). (D) Left atrial diameter (LAD). (E) Combined four‐factor predictive model in the training cohort. (F) Validation cohort performance of the model. (G) Comparison with the APPLE score for predictive performance.

**Figure 4:**
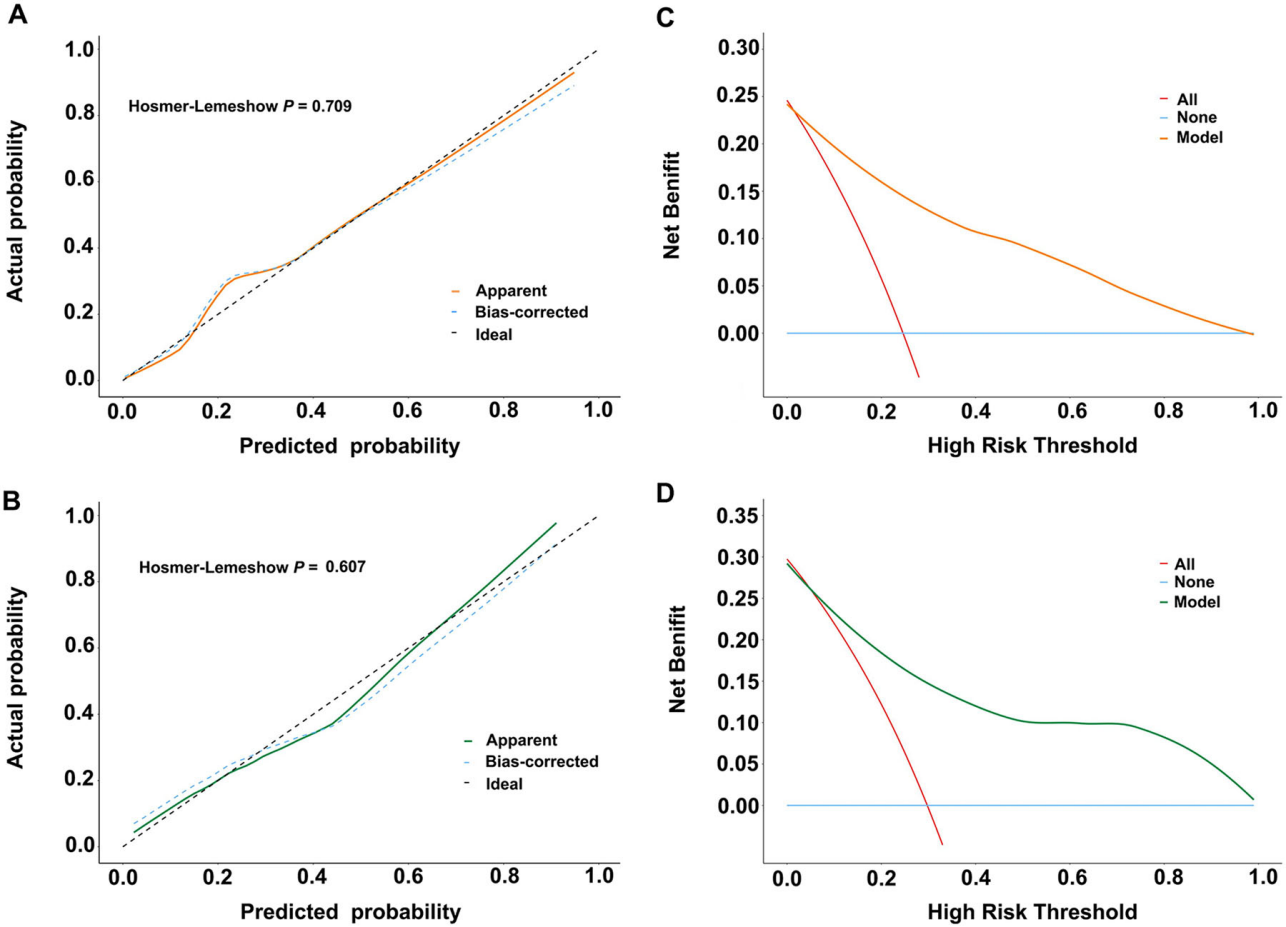
Model performance evaluation. Calibration assessed by the Hosmer−Lemeshow (HL) test in (A) training and (B) validation cohorts. Decision curve analysis (DCA) of clinical net benefit in (C) training and (D) validation cohorts.

ROC analysis identified LAD as the most discriminative single predictor of post‐ablation recurrence (optimal cutoff 41.5 mm, AUC 0.77; 54% sensitivity, 90% specificity), followed by LVZ extent (16.2% cutoff, AUC 0.75), RDW (43.05 fL cutoff, AUC 0.73), and hs‐CRP (4.95 mg/L cutoff, AUC 0.72) (Figure 3A−D). Collinearity diagnostics confirmed the independence of these predictors, with all variance inflation factors (VIF) remaining below 5, eliminating concerns about multicollinearity. The integrated predictive model combining all four parameters achieved superior performance in both training (AUC 0.89; sensitivity 77%, specificity 90%) and validation cohorts (AUC 0.84; sensitivity 78%, specificity 76%) (*p *= 0.3431) (Figure 3E,F). Exploratory analyses by AF type revealed no significant interaction effects. The predictive model demonstrated consistent performance across AF subtypes, with comparable discrimination in paroxysmal (AUC 0.84, 95% CI 0.76−0.92) and non‐paroxysmal (AUC 0.86, 95% CI 0.79−0.93) subgroups (*p *= 0.5522) (Figure S2). Furthermore, the comparative ROC analysis using DeLong's method showed our model achieved significantly higher predictive accuracy (AUC 0.89) compared to the APPLE score (AUC 0.73), with *p* < 0.001 indicating statistically significant improvement in discrimination (Figure 3G). Calibration analysis (Figure 4A,B) showed excellent concordance between predicted and observed outcomes, with apparent and bias‐corrected estimates closely approximating ideal predictions in both training and validation sets. DCA further confirmed the model's clinical utility, demonstrating consistent net benefit across the clinically relevant probability threshold spectrum when compared to default strategies (Figure 4C,D). These results validate our four‐parameter model as a robust, clinically applicable tool for predicting AF recurrence risk following ablation, outperforming existing scores while maintaining generalizability across different AF subtypes. The combination of strong discrimination, accurate calibration, and proven clinical utility supports its potential for guiding post‐ablation management decisions.

### 3.6 Risk Scoring System for Post‐Ablation AF Recurrence

Our novel risk stratification model effectively predicts AF recurrence following ablation by incorporating four key parameters: left atrial LVZ extent, RDW, hs‐CRP, and LAD. Using weighted scores derived from multivariate regression coefficients, we established a five‐tier risk classification system with distinct clinical implications (Table 3). For instance, a 68‐year‐old male with persistent AF scored as follows: LVZ 28% (2 points), RDW 44 fL (6 points), hs‐CRP 8.2 mg/L (3 points), and LAD 48 mm (9 points), totaling 20 points (30%−50% predicted risk). The patient's AF recurrence at 12 months aligns with the intermediate‐high risk prediction. The model distinguished clinical management thresholds: conservative monitoring for scores ≤ 15 versus intensified therapy for scores ≥ 23.

**Table 3:** Risk scoring system for post‐ablation AF recurrence.

Parameter	Range	Points	Total score range	Predicted recurrence probability
LVZ (%)	0−10	0	0−8	< 5%
	11−20	1	9−15	5%−30%
	21−30	2	16−22	30%−50%
	31−40	3	23−29	50%−70%
	41−50	4	≥ 30	> 70%
	51−60	5		
	61−70	6		
RDW (fL)	34−37	0		
	38−41	3		
	42−45	6		
	46−49	9		
	50−54	12		
hsCRP (mg/L)	0.2−5.0	0		
	5.1−10.0	3		
	10.1−15.0	6		
	15.1−20.0	9		
LAD (mm)	32−36	0		
	37−41	3		
	42−46	6		
	47−51	9		
	52−56	12		
	57−60	15		

*Note:* Risk stratification model incorporating electroanatomic substrate characteristics and clinical biomarkers. The total score is calculated by summing individual parameter points.

Abbreviations: hs‐CRP, high‐sensitivity C‐reactive protein; LAD, left atrial diameter; LVZ, low‐voltage zone; RDW, red cell distribution width.

## 4 Discussion

Our study pioneers an integrative predictive model that synergizes four key pathophysiological domains to forecast AF recurrence after catheter ablation. This East Asian cohort study represents the first to combine voltage mapping‐derived fibrosis assessment with hematological and inflammatory biomarkers, addressing a critical gap in personalized management for Asian populations. The model's robust performance (AUC 0.89 in training, 0.84 in validation) stems from its unique ability to capture the multifactorial nature of AF recurrence through distinct yet interconnected biological pathways.

The model's cornerstone lies in direct quantification of atrial fibrosis through LVZ extent, with our ROC‐optimized 16.2% cutoff corroborating established fibrosis thresholds [16]. This electrophysiological parameter reflects the dual arrhythmogenic mechanisms of fibrosis: creating conduction heterogeneity for reentry while increasing pulmonary vein reconnection risk. Notably, Yagishita et al. demonstrated 74% freedom from arrhythmia at 3.1‐year follow‐up after homogenization of fibrotic regions in persistent AF patients, underscoring the therapeutic imperative of substrate modification [17]. This evidence particularly supports aggressive ablation strategies (e.g., posterior wall isolation) in Asian populations, where non‐paroxysmal AF predominates and accounts for 38.0% of cases in our cohort. Notably, a significant proportion of AF patients without detectable LVZs still experience post‐ablation recurrence, suggesting the involvement of alternative pathological mechanisms beyond structural remodeling that warrant further investigation [18].

Beyond erythrocyte volume variability measures, RDW has emerged as a particularly valuable biomarker, offering a cost‐effective window into systemic cardiovascular health. The clinical significance of RDW extends across the AF continuum, from initial development to post‐ablation outcomes, with elevated levels independently predicting incident AF, disease progression to permanent forms, and recurrence following catheter ablation [19, 20, 21]. The pathophysiological underpinnings of this association involve interconnected inflammatory and oxidative stress pathways that simultaneously disrupt erythropoiesis—leading to increased circulating immature erythrocytes and subsequent RDW elevation—and promote endothelial dysfunction, creating a pro‐arrhythmic milieu. Notably, RDW exhibits dynamic predictive utility in the ablation context, where preprocedural elevation correlates with early recurrence while postprocedural reduction independently predicts long‐term success [22, 23]. Our findings reinforce RDW's independent predictive value (OR 1.16, *p* = 0.004) within a comprehensive risk stratification model, supporting its integration into routine clinical assessment given its unique combination of biological plausibility, standardized measurement, and widespread availability in contemporary practice.

The model incorporates hs‐CRP as a sensitive gauge of the pro‐inflammatory milieu that fosters AF recurrence. As a sensitive indicator of systemic inflammation, hs‐CRP elevation reflects an activated biological response to cardiac injury or infection, which under physiological conditions facilitates tissue repair and functional recovery [24]. However, persistent inflammatory activation leads to excessive cytokine production and oxidative stress, creating a pathological substrate for various cardiovascular disorders, including AF [25]. Pro‐inflammatory milieu during the peri‐procedural period has been mechanistically linked to both postoperative AF development and early post‐ablation recurrence [26, 27]. Multiple clinical studies have consistently demonstrated that elevated preprocedural hs‐CRP levels significantly predict increased AF recurrence risk following catheter ablation [28]. Our findings further substantiate this association, identifying hs‐CRP > 4.95 mg/L as an independent predictor of AF recurrence (OR 1.22, *p* = 0.009). Targeted anti‐inflammatory therapy significantly reduced both CRP levels and AF recurrence rates [29, 30, 31]. These collective observations strongly support the incorporation of hs‐CRP assessment into routine clinical practice and highlight the potential benefits of peri‐procedural inflammation modulation in high‐risk patients undergoing AF ablation.

LAD serves as a reliable indicator of atrial structural remodeling, fibrosis progression, and long‐term clinical outcomes in AF [32]. Extensive clinical evidence demonstrates a consistent association between LAD enlargement and post‐ablation recurrence, with thresholds ranging from 40 to 45 mm identified as significant predictors of post‐ablation recurrence [33, 34]. The relationship follows a dose‐dependent pattern, with each 1 mm increase in LAD corresponding to a 7.2% elevation in recurrence risk [35]. Our data confirm these observations, revealing significantly larger LAD values in patients with recurrent AF (*p* < 0.001) and identifying LAD > 41.5 mm as an independent predictor of treatment failure (adjusted OR 1.25, 95% CI 1.09−1.44). These findings collectively highlight the critical role of LAD assessment in risk stratification and the potential benefits of early therapeutic intervention for patients with atrial enlargement.

Current clinical scoring systems for predicting AF recurrence following ablation demonstrate limited discriminative capacity, with AUC values ranging from 0.553 to 0.669 for established models including APPLE, CHA_2_DS_2_‐VASc, and MB‐LATER scores. This constrained predictive performance underscores the need for more robust risk stratification tools in clinical practice [36]. Our novel prediction model demonstrates superior performance through direct electrophysiological substrate assessment using high‐density voltage mapping to quantify LVZ, outperforming conventional indirect markers such as the APPLE score. This comprehensive model uniquely integrates three pathophysiological domains: structural remodeling through LAD measurement, electrical substrate characterization via LVZ quantification, and systemic inflammation/metabolic status assessment using routinely available biomarkers (RDW and hs‐CRP). The model maintains robust predictive accuracy across AF subtypes, with AUC values of 0.84 for paroxysmal and 0.86 for persistent AF.

The developed five‐tier risk stratification system provides clinically actionable guidance. Low‐risk patients may benefit from reduced monitoring intensity with associated cost savings, intermediate‐risk cases warrant intensified rhythm surveillance and potential anti‐inflammatory interventions such as colchicine based on clinical trial evidence, while high‐risk patients require thorough discussion regarding extended substrate modification during potential repeat ablation procedures [37]. This integrated approach represents a significant advancement in precision medicine for AF management, particularly addressing the need for ethnicity‐specific prediction tools in East Asian populations. The incorporation of readily available clinical parameters enhances the model's practical utility and implementation feasibility in routine practice.

While our findings provide valuable insights into AF recurrence prediction, several limitations must be considered when interpreting these results. The single‐center retrospective design may limit the generalizability of our model, as variations in patient populations, ablation techniques, and follow‐up protocols exist across different institutions. This underscores the necessity for external validation through multicenter prospective studies to establish its broader clinical applicability. Additionally, the retrospective design inherently carries risks of selection and information biases that only prospective validation can adequately address. The relatively short follow‐up duration precludes assessment of very late recurrences, warranting extended observation periods of 3−5 years in future investigations.

In conclusion, this study proposes a novel integrated predictive tool that combines electrophysiological substrate characteristics, inflammatory markers, and structural remodeling parameters to provide clinically actionable risk stratification for AF recurrence in Asian populations. The model exhibits a high degree of predictive accuracy while maintaining cost‐effectiveness, thus aligning with the requirements of precision medicine and healthcare economics in clinical practice. However, further validation through prospective multicentre cohorts is warranted to confirm its generalizability, and exploration of risk score‐guided personalized therapeutic pathways represents an important direction for future research. Addressing these limitations in future research will be essential to refine the model's reliability and optimize its clinical implementation.

## Author Contributions

Juan Zhang conceived and designed the study. Ying Han and Jingzhe Liu performed data collection and analysis. Xiaobo Liu and Hanyue Zheng contributed to data processing. Juan Wang and Juan Zhang drafted the manuscript. All authors critically reviewed and approved the final version.

## Conflicts of Interest

The authors declare no conflicts of interest.

## Supporting information

Supporting data1. AF.

Supporting Figure 2. ROC‐AF subtypes.

Supporting Figure1. Patient flowchart.

Supporting Table 1. Training set and validation set.

## Data Availability

All data underlying the findings described in this manuscript are fully available without restriction in the Supporting Information files accompanying this article.
