# Supplementary figures and images for: A Novel Clinical Score Integrating Low‐Voltage Zones and Biomarkers Predicts Atrial Fibrillation Recurrence Post‐Ablation
Source: Clin Cardiol. 2025 Nov 12;48(11):e70218. doi: 10.1002/clc.70218 (PMC12611274; doi:10.1002/clc.70218)

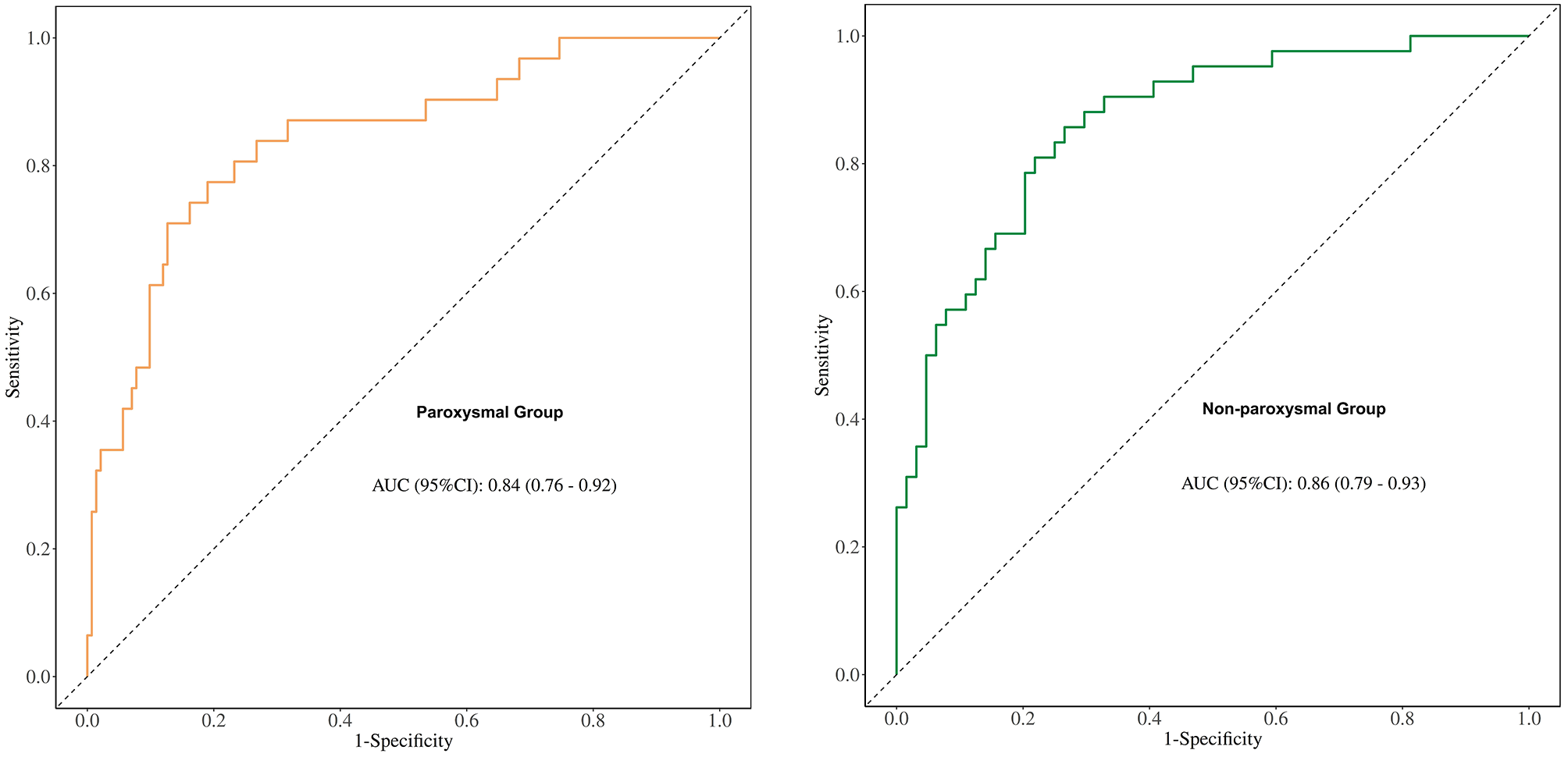

Supplement: Supplementary file 2 — Supporting Figure 2. ROC‐AF subtypes. [file CLC-48-e70218-s004.tif]

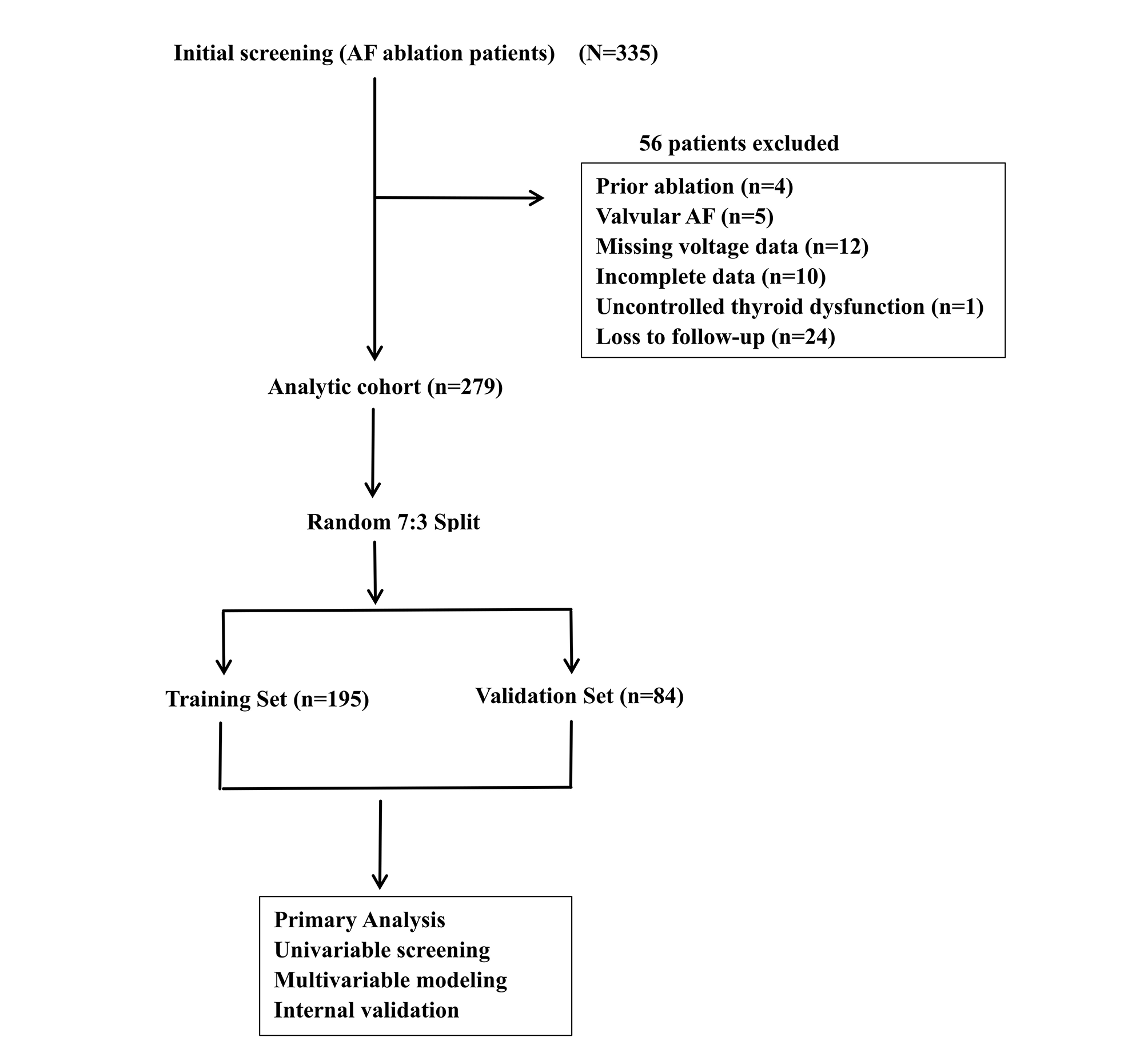

Supplement: Supplementary file 3 — Supporting Figure1. Patient flowchart. [file CLC-48-e70218-s003.tif]
