# Supplementary material for: A Novel Clinical Score Integrating Low‐Voltage Zones and Biomarkers Predicts Atrial Fibrillation Recurrence Post‐Ablation
Source: Clin Cardiol. 2025 Nov 12;48(11):e70218. doi: 10.1002/clc.70218 (PMC12611274; doi:10.1002/clc.70218)
Supplement: Supplementary file 4 — Supporting Table 1. Training set and validation set. [file CLC-48-e70218-s002.docx]

Table 1. Baseline characteristics of patients in the training set and validation set.

| **Characteristics** | **Total (n = 279)** | **Validation (n = 84)** | **Training (n = 195)** | ***P*-value** |
| --- | --- | --- | --- | --- |
| Recurrence, n(%) | 73 (26.16) | 25 (29.76) | 48 (24.62) | 0.370 |
| Age, (years) | 66.00 (59.00, 72.00) | 65.50 (58.75, 71.25) | 66.00 (59.50, 72.00) | 0.708 |
| Female, n(%) | 121 (43.37) | 37 (44.05) | 84 (43.08) | 0.881 |
| Non-paroxysmal AF, n (%) | 106 (37.99) | 31 (36.90) | 75 (38.46) | 0.806 |
| AF duration, months | 15.00 (9.00, 30.00) | 14.00 (11.50, 35.00) | 18.00 (8.00, 30.00) | 0.898 |
| Smoking, n(%) | 96 (34.41) | 34 (40.48) | 62 (31.79) | 0.161 |
| Drinking, n(%) | 88 (31.54) | 27 (32.14) | 61 (31.28) | 0.887 |
| Hypertension, n(%) | 153 (54.84) | 46 (54.76) | 107 (54.87) | 0.987 |
| Diabetes mellitus, n(%) | 54 (19.35) | 11 (13.10) | 43 (22.05) | 0.082 |
| Hyperlipidemia, n(%) | 44 (15.77) | 17 (20.24) | 27 (13.85) | 0.179 |
| Stroke, n(%) | 42 (15.05) | 10 (11.90) | 32 (16.41) | 0.334 |
| Coronary heart disease, n(%) | 166 (59.50) | 47 (55.95) | 119 (61.03) | 0.428 |
| LAD（mm） | 42.00 (39.00, 45.00) | 41.00 (39.00, 44.00) | 42.00 (39.50, 45.00) | 0.342 |
| LVEDD（mm） | 47.00 (45.00, 50.00) | 47.00 (45.00, 50.00) | 47.00 (45.00, 50.00) | 1.000 |
| LVESD（mm） | 31.00 (29.00, 34.00) | 31.00 (30.00, 34.00) | 31.00 (29.00, 34.00) | 0.236 |
| LVEF（%） | 0.60 (0.57, 0.64) | 0.60 (0.56, 0.64) | 0.61 (0.57, 0.64) | 0.230 |
| Erythrocytes (10^9/L) | 4.61 ± 0.58 | 4.64 ± 0.57 | 4.60 ± 0.59 | 0.646 |
| Hemoglobin (g/L) | 141.01 ± 17.11 | 142.70 ± 17.24 | 140.28 ± 17.05 | 0.278 |
| RDW (fL) | 43.30 (40.90, 45.10) | 42.75 (40.88, 44.95) | 43.50 (40.95, 45.20) | 0.337 |
| Leukocytes (10^9/L) | 6.10 (4.98, 7.14) | 6.29 (5.19, 7.33) | 6.00 (4.82, 7.04) | 0.217 |
| Neutrophils (10^9/L) | 3.58 (2.91, 4.51) | 3.76 (2.95, 4.74) | 3.53 (2.84, 4.47) | 0.214 |
| Lymphocytes (10^9/L) | 1.71 (1.34, 2.04) | 1.72 (1.34, 2.07) | 1.71 (1.35, 2.01) | 0.561 |
| Monocytes (10^9/L) | 0.45 (0.35, 0.55) | 0.48 (0.39, 0.54) | 0.43 (0.35, 0.56) | 0.097 |
| Platelets (10^9/L) | 212.05 ± 47.91 | 212.32 ± 48.48 | 211.93 ± 47.78 | 0.950 |
| TG（mmol/L） | 1.19 (0.90, 1.60) | 1.24 (0.90, 1.67) | 1.14 (0.91, 1.56) | 0.304 |
| TC（mmol/L） | 3.98 (3.45, 4.70) | 4.05 (3.47, 4.89) | 3.96 (3.45, 4.64) | 0.429 |
| LDL-C（mmol/L） | 2.26 (1.71, 2.79) | 2.30 (1.89, 2.83) | 2.24 (1.65, 2.79) | 0.194 |
| HDL-C（mmol/L） | 1.14 (0.96, 1.37) | 1.16 (0.91, 1.36) | 1.12 (0.97, 1.42) | 0.778 |
| Albumin (g/L) | 42.55 ± 3.47 | 42.69 ± 3.50 | 42.49 ± 3.47 | 0.667 |
| Hcy (μmol/L) | 15.80 (12.10, 17.95) | 16.40 (12.17, 18.20) | 15.70 (12.05, 17.85) | 0.428 |
| Cys-C (mg/L) | 1.16 (1.03, 1.31) | 1.18 (1.04, 1.35) | 1.15 (1.02, 1.29) | 0.272 |
| hs-CRP (mg/L) | 2.60 (1.20, 5.15) | 2.85 (1.37, 5.50) | 2.57 (1.20, 5.05) | 0.225 |
| Uric acid (μmol/L) | 327.00 (277.00, 405.00) | 329.00 (278.50, 386.25) | 327.00 (276.50, 409.00) | 0.870 |
| Creatinine (μmol/L) | 70.00 (62.00, 79.00) | 73.00 (64.00, 82.25) | 68.00 (62.00, 77.00) | **0.019** |
| eGFR (ml/min) | 89.10 (70.25, 103.30) | 83.70 (72.15, 98.35) | 92.10 (69.75, 104.85) | 0.107 |
| NT-proBNP（pg/ml） | 509.00 (302.50, 872.10) | 543.80 (307.35, 949.08) | 506.90 (295.90, 856.25) | 0.959 |
| D-dimer（ug/ml） | 0.27 (0.17, 0.43) | 0.26 (0.15, 0.41) | 0.28 (0.17, 0.43) | 0.634 |
| Fibrinogen (g/L) | 2.93 (2.61, 3.29) | 3.03 (2.72, 3.40) | 2.86 (2.54, 3.24) | **0.014** |
| Total left atrial area(cm²) | 193.80 (167.80, 224.90) | 187.25 (167.73, 221.50) | 197.80 (168.00, 225.75) | 0.498 |
| Percentage of abnormal voltage( %) | 19.09 (9.68, 29.78) | 19.25 (10.76, 28.81) | 19.09 (9.63, 29.96) | 0.941 |
| Abnormal voltage |  |  |  | 0.442 |
| ＜10%, n(%) | 77 (27.60) | 20 (23.81) | 57 (29.23) |  |
| 10-20%, n(%) | 70 (25.09) | 18 (21.43) | 52 (26.67) |  |
| 20-30%, n(%) | 68 (24.37) | 24 (28.57) | 44 (22.56) |  |
| ＞30%, n(%) | 64 (22.94) | 22 (26.19) | 42 (21.54) |  |
